# Role of emodin to prevent gastrointestinal cancers: recent trends and future prospective

**DOI:** 10.1007/s12672-025-02240-9

**Published:** 2025-04-05

**Authors:** Falak Thakral, Bhairav Prasad, Rippin Sehgal, Saurabh Gupta, Ujjawal Sharma, Bikram Jit Singh, Bunty Sharma, Hardeep Singh Tuli, Shafiul Haque, Faraz Ahmad

**Affiliations:** 1 Department of Bio-Sciences and Technology, Maharishi Markandeshwar Engineering College, Maharishi Markandeshwar (Deemed to Be University), Mullana, Ambala, India; 2 Department of Biotechnology, Chandigarh Group of Colleges, Landran, Mohali, Punjab India; 3 Department of Biotechnology, Ambala College of Engineering and Applied Research, Devsthali, Ambala, Haryana 133101 India; 4 Mata Gujri College, Fatehgarh Sahib, Punjab India; 5 Department of Human Genetics and Molecular Medicine, Central University of Punjab, Bhatinda, 151001 India; 6 Mechanical Engineering Department, MM Engineering College, Maharishi Markandeshwar (Deemed to Be University), Mullana, Ambala, Haryana 133207 India; 7 Department of Biotechnology, Graphic Era (Deemed to Be University), Dehradun, Uttarakhand India; 8 Department of Nursing, College of Nursing and Health Sciences, Jazan University, Jazan-45142, Saudi Arabia; 9 School of Medicine, Universidad Espiritu Santo, Samborondon, 091952, Ecuador; 10 Department of Biotechnology, School of Bio Sciences and Technology (SBST), Vellore Institute of Technology, Vellore, 632014 India

**Keywords:** Emodin, Gastrointestinal cancers, Phytochemicals, Anticancer properties, Nanoparticles

## Abstract

Gastrointestinal malignancies are responsible for approximately 35% of all cancer-related deaths, underscoring the critical need to explore pharmacologically active molecules for chemoprevention. Emodin (1,3,8-trihydroxy-6-methylanthraquinone), a natural compound derived from traditional Chinese and Japanese medicine, has recently garnered significant attention for its potential anticancer properties. Emodin exerts its chemoprotective effects through a combination of antioxidative, anti-inflammatory, and anti-proliferative mechanisms. Research indicates that emodin inhibits cancer metastasis, disrupts cell cycle progression, and impairs cancer cell survival. These effects are mediated through the activation of the p38 MAPK/JNK1/2 signaling pathway, the upregulation of pro-apoptotic factors such as Bax/Bcl-2 and caspases, and the enhancement of reactive oxygen species (ROS) levels (Supplementary Fig. 1). To optimize emodin’s therapeutic potential, it is crucial to further investigate its underlying mechanisms of action and develop advanced nano-targeted delivery systems to enhance its bioavailability. This review highlights emodin’s promise as a chemopreventive agent for gastrointestinal cancers and emphasizes its potential for development into a novel clinical formulation.

**Supplementary Information:**

The online version contains supplementary material available at 10.1007/s12672-025-02240-9.

## Introduction

One of the most prevalent cancers in the world today is gastrointestinal cancer (GI). Among all cancers, GI ranks second in terms of mortality rates [1]. GI causes major malignances over worldwide and which not fully elucidated. There are six major GI cancers identified by International classification of diseases (ICD 10th revision). The six major classes of GI are esophagus (EC), Gastric (GC), Liver (LC), Pancreatic (PC), colon and rectal cancer. Over 3 million deaths and 4.1 million new cases are caused by these tumours year worldwide [2]. Each kind of GI cancer accounts for roughly 700,000 deaths annually [3]. The most prevalent kind of tumours are GI malignancies, which often afflict both sexes. Cancer has become a more common cause of mortality and difficulty due to population expansion and ageing as well as inequality in cancer control. These cancers are currently being treated with cytotoxic chemotherapy, radiotherapy, immunotherapy, and/or surgery [4]. There hasn't been much progress in addressing the prevalent off-target effects and acquired drug resistance that these treatment paradigms bring with them, despite the fact that they have definitely increased survival. Utilising natural substances has demonstrated potential in reducing the growth of tumours and mitigating side effects related to conventional chemotherapy treatments. Many naturally occurring substances have extremely wide pleiotropic effects, which makes them appealing candidates to target the various pathways causing cancer and adverse drug reactions [5].

Emodin, an anthraquinone derived from Chinese herbs, has shown potential as a natural anti-cancer agent in pre-clinical trials for the treatment of several types of cancer, including those of the liver, pancreatic, gallbladder, stomach, colon, and lung [6]. Chinese medicinal herbs contains emodin, which is utilized in traditional Chinese and Japanese medicine, include *Rheum palmatum, Polygonum cuspidatum,* and *Cassia obtusifolia* [7]. Emodin has been shown by modern pharmacology to have a number of beneficial benefits, including anti-inflammatory, anti-tumor, anti-oxidant, laxative and hypoglycemic actions [8]. Emodin’s anti-tumor activities are achieved through altering several hallmarks of cancer, including as invasion and metastasis, tumour angiogenesis, epithelial-mesenchymal transition (EMT), cell growth (i.e., promotion of apoptosis, reduction of proliferation, and changed cellular redox status). It has been determined that emodin modulates a number of oncogenic signalling pathways and molecules, including as HIF-1α, NF-κB, HER-2, STAT3, AKT/mTOR, Wnt, p38/p53/Puma, and VEGFR-2 [9].

## Chemistry and pharmacokinetics

**Fig. 1:**
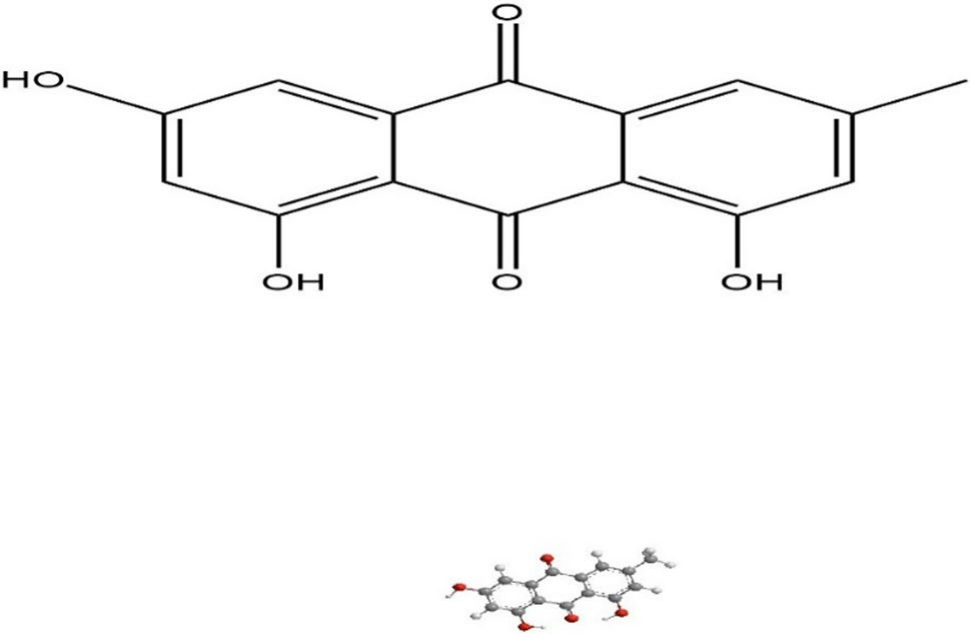
Emodin structure generated by chemdraw

Emodin is a natural compound found in several plants, known as an anthraquinone. It has a variety of beneficial effects on health, offering several pharmacological activities [10]. Structurally Emodin is 1,3,8-trihydroxy-6-methylanthraquinone (Fig. 1) [11].

A naturally occurring anthraquinone derivative called emodin has attracted a lot of interest due to its possible therapeutic benefits, especially when it comes to GI. [12]. Pharmacokinetically, emodin exhibits a complex profile characterized by moderate absorption, extensive metabolism, and variable bioavailability [13]. Upon oral administration, emodin is absorbed in the small intestine, but its bioavailability is often limited by poor solubility and rapid metabolism [14]. In the liver, emodin is extensively metabolised in phases I and II, producing a number of metabolites, primarily glucuronides and sulfates, which are excreted via bile and urine [10]. The enterohepatic recirculation of emodin and its metabolites contributes to a prolonged half-life, enhancing its therapeutic window [15]. In the context of GI cancers, the distribution of emodin to the gastrointestinal tract is particularly advantageous, allowing for direct interaction with cancer cells in the stomach, colon, and rectum [16]. Studies have shown that emodin can induce apoptosis, proliferation inhibition, and suppress metastasis of cancer cells through various molecular pathways, including the inhibition of NF-κB, PI3K/Akt [17], and Wnt/β-catenin signaling [18]. Despite these promising pharmacological activities, the clinical application of emodin is hampered by its pharmacokinetic limitations [19]. Strategies such as nanoparticle delivery systems and structural modifications are being explored to enhance its solubility, stability, and bioavailability [20]. Overall, understanding the pharmacokinetics of emodin is crucial for optimizing its therapeutic efficacy against GI cancers, paving the way for its potential incorporation into clinical practice.

## Biosynthesis of emodin

**Fig. 2:**
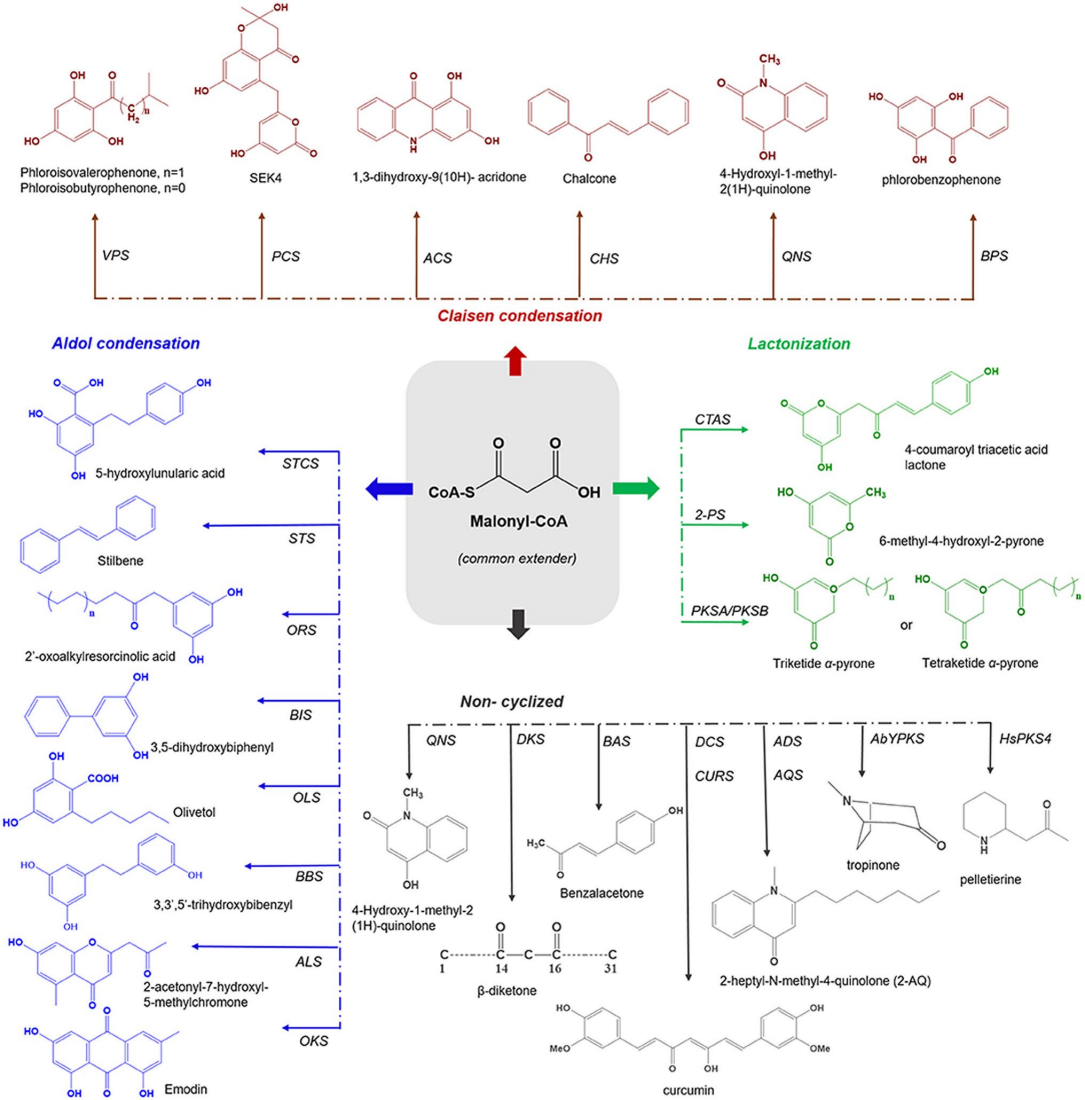
Process of Biosynthesis of Emodin [24]

Emodin, a naturally occurring derivative of methylanthraquinone, is widely distributed among higher plants, particularly within the families Polygonaceae, Fabaceae, and Rhamnaceae. Its presence has also been documented in various fungal genera, including *Aspergillus*, *Penicillium*, and *Cladosporium* [21]. This compound is commonly found in plants alongside other structurally related anthraquinones, including rhein, chrysophanol, aloe-emodin, and physcion. The biosynthesis of anthranoids in plants is believed to involve octaketide synthase (OKS), a member of the type III polyketide synthase (PKS) enzyme superfamily. PKSs generate diverse polyketide scaffolds, including chalcone, stilbene, acridone, curcumin, and isocoumarin [22]. Type III PKSs are homodimers with subunit masses of 40–45 kDa. Structurally, type III PKSs are compact homodimeric proteins, with each monomer harboring an independent active site featuring a catalytic triad of cysteine (Cys), histidine (His), and asparagine (Asn). They catalyze the stepwise condensation of acetyl units from malonyl-CoA decarboxylation onto a starter CoA ester, resembling fatty acid biosynthesis. Their functional diversity arises from variations in starter molecule selection, the number of malonyl-CoA condensations, and cyclization mechanisms [23]. Type III PKSs employ a straightforward strategy, priming with a monocarboxyl-CoA starter substrate followed by iterative decarboxylative condensations with dicarboxyl-CoA extenders to form a poly-β-keto intermediate. This intermediate can be cyclized or released as a linear product. Their versatility lies in the ability to accept diverse starter and extender substrates, enabling various condensation and elongation reactions explained in Fig. 2. OKS uses acetyl-CoA as a starter, sequentially condensing it with seven malonyl-CoA molecules to produce a linear octaketide intermediate. Oxidation of the central cyclohexadienone ring in emodin anthrone forms emodin.

## Major gastrointestinal cancer

According to numerous reports, cancer is the leading cause of death and the largest obstacle to raising life expectancy in both developed and developing countries. Numerous plants naturally contain emodin (1,3,8-trihydroxy-6-methylanthraquinone), an anthraquinone derivative with a wide range of biological activities, such as hepatoprotective, antibacterial, anti-inflammatory, and anticancer effects. Regarding a number of cancer types, such as colon cancer, breast cancer, cervical cancer, leukaemia, lung cancer, ovarian cancer, liver cancer, and pancreatic cancer, Emodin provides accurate results [25]. Gastrointestinal (GI) cancers are one of the main cancers worldwide and pose a serious threat to human health. GI cancer account for 26% cancer incidence globally and 35% of all cancer related deaths. According to a 2018 survey, there were 3.4 million GI cancer deaths and 4.8 million new cases globally in that year [26].

- A. Esophageal cancer

**Fig. 3:**
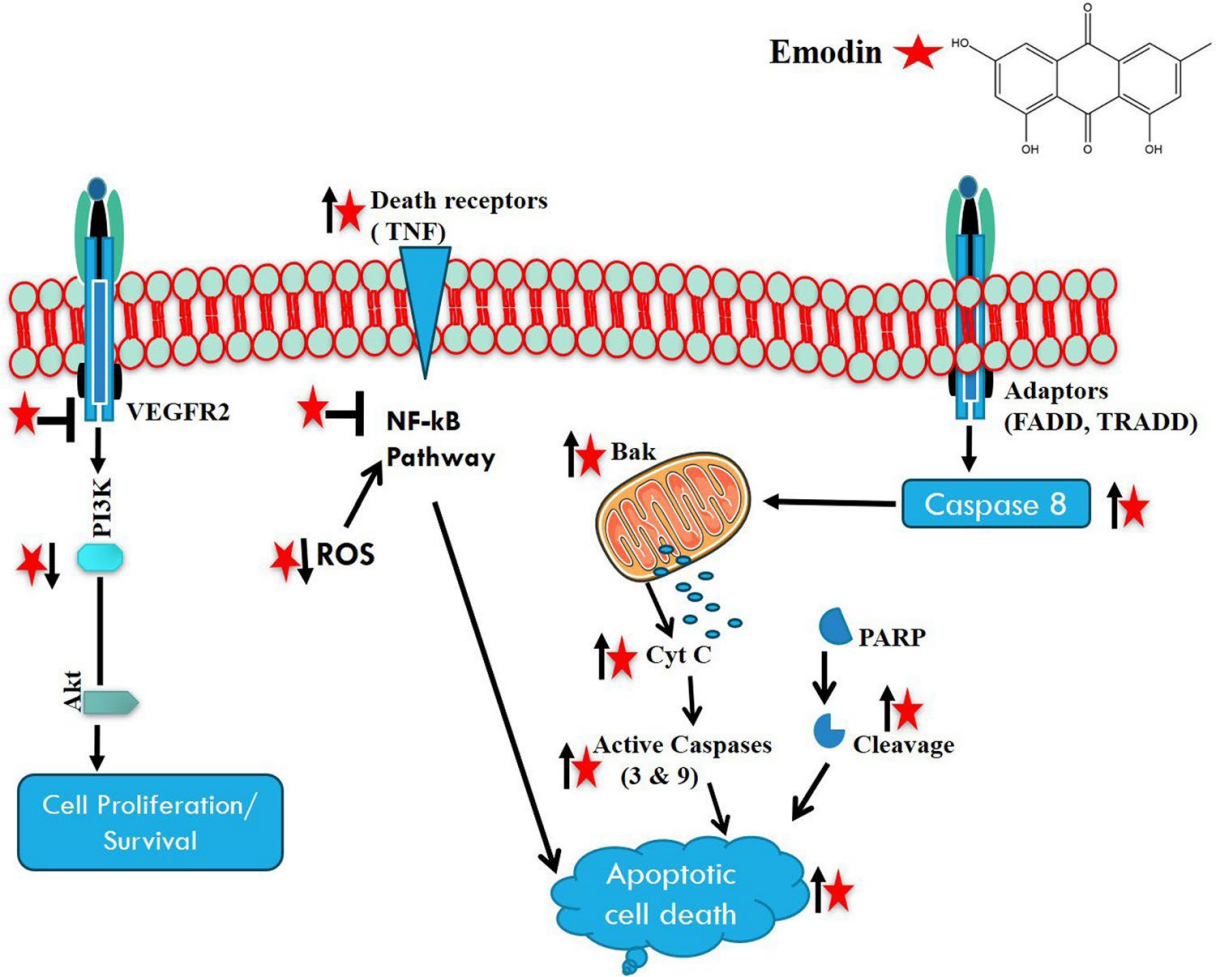
Illustration of emodin mediated apoptosis activation. Emodin can activate the apoptosis of cancer cells by activating intrinsic pathways via increasing the expression of cytochrome C, caspa-9, -3, and via changing the mitochondrial membrane potential; and extrinsic pathway via increasing the expression of death receptor, caspase 8, Bak protein. Emodin can also inhibit NF-kB pathway and decreased ROS production to activate apoptotic cell death. Further, it also inhibits the dimerization of VEGFR2 receptors and inhibit downstream PI3, AKT pathway

Esophageal cancer (EC), a highly invasive cancer, was the eighth most common cancer and the sixth leading cause of cancer-related death in 2020 [27]. EC has high mortality rate due to its poor prognosis and remain asymptomatic at early stage and presenting common symptoms including Dysphagia alone or with unintentional weight loss. Adenocarcinomoa and squamous cell carcinoma are the common esophageal cancer and account more than 95% of esophageal cancer [28]. Emodin, has diverse biological activity and at first it was thought that it has ability to inhibit protein tyrosine kinase p65*lck*, but now results showing it can interact with many cellular targets and can modulate or inhibit mammalian cell cycle in oncogene over expressed cell suggesting the compound having anti-cancerous property [29]. AKT and ERK are signal regulated kinase and observed in many cancerous cells and play important role in cell differentiation, proliferation, and survival. Additionally involved in the TE1cell of esophageal cancer are these kinases, AKT and ERK. Aloe-emodin, which is found in aloe latex, has the capacity to dose-dependently inhibit the phosphorylation of AKT and ERK, suppressing their function and TE1 cell proliferation in the S-phase of the cell cycle, thereby halting the progression of cancer. Therefore, Aloe-emodin can be used in esophageal cancer therapy [30]. Similarly, in another study the anti-cancer effect of emodin was evaluated against human esophageal carcinoma cell line ECA109. The results revealed that the emodin induces cell apoptosis in ECA109 cell line and inhibit cell invasion by modulating and down regulating the activation and expression of MMP2, Bax/Bcl-2 and caspase-3 involving in esophageal cancer [31]. Emodin has been shown in a related study to cause apoptosis and inhibit the growth of EC9706 cells. When combined with ciplatin, emodin significantly raises intracellular ROS, which improves proapoptotic effects and can be used to treat esophageal cancer [32] (Fig. 3).

- B. Gastric cancer

The most prevalent kind of stomach cancer, gastric cancer (GC), has a variety of histological presentations and is still difficult to diagnose and treat. The most prevalent type of gastric cancer, tubular adenocarcinoma, accounts for over half of all cases and has been reported to be as high as 72% in South Korea [33, 34]. Emodin also effectively used in gastric cancer prevention and treatment. RhoA is a signaling molecule and play important role in gastric cancer and regulating variety of cellular process for the cancer progression. It has been seen that emodin induced reactive oxygen species generation which, inhibit RhoA activation and sensitized gastric cell carcinoma to transform them into anoikis (inappropriate cell–matrix interactions) thus inhibiting the proliferation of gastric cancer [35]. Helicobacter pylori is a gram-negative pathogenic bacteria colonize in the 50% human population intestine worldwide and lead to many infections including gastric ulcer, Chronic gastritis, gastric cancer and many more. Eradication of H. pylori from intestine could prevent the development and progression of these diseases [36]. Aloe-emodin reported to successfully inhibit the colonization of this bacteria in the intestine and destroy the biofilm formation by H. pylori, thus can be used for the clinical treatment of H. pylori and its associated disease [27]. It has also reported that emodin use mitochondrial mediate apoptosis (MMA) mechanism to inhibit GI cancer. The pro- and anti-apoptotic proteins in the mitochondrial membrane are primarily balanced to control MMA. Interestingly, the mitochondria of GI cancer cells have been found to contain emodin at concentrations of 50 and 100 µM [37]. The emodin trigger the mitochondria of GI cancer cells to disrupt mitochondrial membrane potential by signaling molecules Bax, Bak PARP and triggers the release of cytochrome c to activate caspase -9 and 3 culminating into apoptosis [38]. Emodin along with 5FU is commonly used to cure gastric cancer. Their combination exerts a synergistic effect on inhibiting the proliferation of gastric cell cancer and promotes cell apoptosis [32]. It has been also evaluated that alone emodin show weak inhibitory activity on cell proliferation but when it combined with 5FU in a dose dependent manner it significantly increase its impact and restricted cell growth, reduce cell division index, decrease the size of many cells of gastric cancer cell within 48 h of treatment and also allow to differentiate cell line boundaries [39] (Fig. 3).

- C. Colorectal cancer

In both sexes, colorectal cancer (CRC) is the third most common cause of cancer-related deaths and the third most common type of cancer diagnosed in the United States. It accounts second rank in cancer related death in men younger than 50 years. Greater than ½ cases of all deaths are associated with modifiable risk factors such as sedentary and unhealthy life style (16%), smoking (11%), obesity (5%), unhealthy diet (29%) and excessive alcohol consumption (13%) [40]. Emodin also effectively used to treat CRC, as it has ability to inhibit the proliferation of CRC cells by targeting VEGFR2 (vascular endothelial growth factor receptor 2) a signaling pathway. The expression of VEGFR2, p-AKT, and PI3K is significantly high in CRC cells and thus favoring cell proliferation and cancer progression. Emodin bound the hydrophobic pocket of VEGFR2 and inhibiting the dimerization of VEGFR2 and lowers the expression of p-AKT and PI3K in HCT116 cells in vivo and tumor bearing mice [41]. Another study reported that emodin regulating Bcl-2/Bax ratio and cytochrome c and can inhibit the proliferation of LOVO colorectal cancer [42]. The ROS/p38/p53/Puma signalling pathway can be regulated by emodin to cause apoptosis in human colorectal cancer cells SW620 and SW480 [75], and it can also suppress Wnt signalling in these cells [74]. However, the exact way that emodin treats colorectal cancer is unknown. ACSL4 has direct role in CRC development by increasing the expression of VEGFR1 and VEGFR2. Emodin induced pathway exert a signaling pathway that suppress the activation of ACSL4 thus the expression of VEGF secretion system and inhibiting CRC proliferation [41, 43] reported that the emodin administration of (40 or 80 mg/kg in AOM/DSS and 80 mg/kg in ApcMin/ +) orally thrice per week shows preventive effect. It also reduces the size and polyp count both in rodent models also show lower protumerogenic M2 like macrophages in colon microenvironment and also there is reduced ratio of M2/M1 macrophage within the colon (P < 0.05). Ferroptosis is a novel apoptosis method associated with iron-dependent lipid peroxidation and a promising strategy in cancer treatment. It has been linked that Emodin induce ferroptosis and suppressed tumor growth in xenograft mouse model. Additionally, it was determined that by deactivating the NF-κb pathway in CRC cells, emodin caused ferroptosis via NCOA4-mediated ferritinophagy [44] (Fig. 3).

- D. Pancreatic cancer

Pancreatic cancer (PC) is one of the life threatening cancer and leading cause of cancer linked death in United States [45]. The exact cause of PC is not clear but many modifiable and non-modifiable risk factors are linked with PC. The modifiable risk factors include obesity, infection, dietary factors, alcohol consumption, smoking, pancreatitis and socieo- economic status, while the non-modifiable risk factors include gender, age, ABO blood group, ethnicity, microbiota, Diabetes mellitus, genetic susceptibility and family history [46]. A range of malignant tumours are directly inhibited by emodin. By preventing EMT (epithelial mesenchymal transition) in tumour cells and inducing tumour cell apoptosis in pancreatic carcinoma cells, micro-RNA-1271 (miRNA 1271) exhibits broad tumor-suppression activity. It also inhibits the growth and metastasis of a number of malignant tumour cells. Through the inhibition of tumour cells’ EMT and the induction of tumour cell apoptosis, miR 1271 has a broad tumor-suppression effect. Emodin show positive impact on miR 1271 expression thus supporting the tumor cell apoptosis via EMT regulatory mechanism [42]. Similarly, over expression of EGFR is closely associated with tumor formation, their progression and deterioration and liked with 90% of the pancreatic cancer. However, drug targeting EGFR inhibitor is the easy way to control disease but drug resistance of EGPR inhibitor making treatment challenging. Though. Emodin surprisingly. Enhanced the anti-cancer effect of EGPR inhibitor on pancreatic carcinoma, it also promote afatinib-induced apoptosis by blocking the STAT 3 signaling pathway [47]. Emodin-loaded lipid nano-capsules (M-CS-E-LNC)—mannose-conjugated chitosan-coated emodin nano-capsules—were used to treat severe acute pancreatitis. The carnitine palmitoyltransferase 1 protein was able to be up-regulated by the emodine nanocapsule, which ultimately facilitated intracellular long-chain fatty acid transport. This led to the M2 phenotype polarization of macrophages and a decrease in the amount of inflammatory mediators in macrophages [48]. Emodin based a novel drug delivery system was formulated to treat acute pancreatitis in mouse model. A membrane-coated UiO-66-NH2 that has been loaded with emodin (MVs-UiO-ED). MVs-UiO-ED nanoparticle was found to decrease serum levels of lipase and α-amylase, two important markers of the severity of pancreatitis. Additionally, histopathological analyses demonstrated that MVs-UiO-ED reduced pancreatic tissue damage, and the author's findings support the treatment of acute pancreatitis [49] (Fig. 3).

- E. Liver cancer

Liver is the principal organ in body involve in detoxification of toxic chemicals, pathogens and metabolic waste. The organ is in continuous exposure to viruses, alcohol, bio-transformed metabolites and cholestasis which sometime lead to liver inflammation and liver injury. When injury sustained for long time it culminating into chronic liver disease [50]. Emodin, possess antiproliferative and apoptosis effects in many cancer cell lines. In liver cancer, emodin induces apoptosis in HepG2 cells and arrest the cells in their G1 phase. Additionally, it activates CASPASE -8 and CASPASE-9 and causes cytochrome c to be released from mitochondria into the cytosol. Additionally, it raises p53 protein levels and lowers NF-κB/p65 levels in HepG2 cells; these two regulators may be important in HepG2 cell apoptosis induced by emodin [51]. Emodin shows promising role in treating HCC by suppressing the growth and movement of HCC cell and also targeting CD +  + hepatoma cells by inhibiting its proliferation [52]. In another study it has been reported that the emodin play multi role in liver disease. It is able to reduce collagen synthesis, inhibiting oxidative stress thus prevent liver fibrosis, inhibiting TGF-β/Smad pathway and HSCs proliferation and promoting HSCs apoptosis. It also regulates the lipid metabolism in liver thus regulate the oxidation of lipid and cholesterol synthesis and prevent nonalcoholic fatty liver. Emodin also play significant role in inhibiting liver cancer by inducing mitochondrial apoptosis pathway in hepatocellular carcinoma cells [53]. According to another study, emodin suppresses transcriptional activity and sterol regulatory element-binding protein-2, which in turn suppresses cholesterol biosynthesis and oncogenic protein kinase B (AKT) signalling. STAT3, or signal transducer and activator of transcription 3, and oncogenic transcription factors are rendered inactive by regulated cholesterol synthesis and oncogenic AKT signalling. Emodin’s sensitising effect on sorafenib-based therapy for HCCs was also assessed, and it was found to significantly enhance sorafenib’s anti-cancer effect in liver cell cancers, including HepG2, Hep3B, Huh7, SK-HEP-1, and PLC/PRF5. Furthermore, sorafenib and emodin increased apoptosis in HCCs and cell cycle arrest in the G1 phase in a synergistic manner [54] (Fig. 3). Various studies focusing on the anticancer effect of emodin have been summarized in Tables 1 and 2.

**Table 1:** Anticancer potential of emodin in various types of cancer cell lines and animals

S.no	Type of cancer	Cell line/animals	Concentration	Mechanism	Reference
1	Hepatic carcinoma	HepG2 cells	20–200 µM	Reduces wadburg effect by ↓ HKII, PKM2, LDHA, limiting energy supply, ↑ ROS production, ↑ mitochondrial damage and apoptosis	[55]
2	Breast cancer	SKBR3 cells	10–50 µM	↑ apoptosis, ↑mRNA expression of caspase 3,8, 9 and Bax, ↓ Bcl2 expression	[56]
3	Breast cancer	C57BL/6 Mice	40 mg/Kg	↓ TGF- β1 signalling pathway, inhibit transcription factor to EMT and CSC	[57]
4	Lung cancer	NCSLC cells	25–75 µM	↓ redox metabolism, ↑ROS levels, ↑ DNA damage specific to cancer cells and leading to senescence	[58]
5	Lung cancer	NCSLC cells	–	↓ sPLA2-IIa and NF-κB pathways, inhibited mTOR & AKT, activated AMPK, ↑apoptosis, cell cycle arrest & ROS production	[59]
6	Ovarian cancer	SK-OV-3, A2780, PA-1	2.5–80 µM	Inhibited EMT by ↓ N cadherin & vimentin and ↑ E-cadherin	[60]
7	Cervical cancer	HeLa cells	100 µM	Induction of mitotic catastrophe, inhibited mitosis mainly in metaphase, G2/M phase, ↑ apoptosis	[61]
8	Breast cancer	EO771 & 4 T1	50 µM	Inhibited cellular proliferation & migration by ↓ CD155	[62]
9	Bladder cancer	T24 & 5637	20–80 µmol/L	↓ cellular growth, invasion & expression of Notch 1, Jagged 1, VEGF, VEGFR2, MMP2	[63]
10	Glioma	C6 & U87-MG	30–300 µM	Deactivates phosphorylated AKT, ceases nuclear translocation of NF-κB, ↓ β- Catenin, ↑ N-cadherin	[64]
11	Glioma	C6	–	↓ ERK1/2, ↑ Caspase dependent autophagy	[65]
12	Glioma	U87-MG	20–40 µM	Arrest cell cycle in S & G2M phase, ↑ PARP and Lamin, ↓pAKT phosphorylation	[66]
13	Prostate cancer	PC3	20–80 µg/ml	Cell cycle arrest in S & G2/M phase, ↑ Notch 1, ↓ Jagged1, VEGF & bFGF	[67]
14	Prostate cancer	PC3	10–40 µmol/L	↓Androgen-dependent transactivation, induces androgen receptor degradation	[68]

**Table 2:** Anticancer effects of emodin on various GI cancers

S.No	Type of cancer	Subjective model	Physiological effects	Mechanism of action	References
1	Esophageal cancer	ECA109 cells	↑Apoptosis	↓ MMP-2, Bcl-2 & ↑expression of Bax and Caspase	[31]
2	Esophageal cancer	TE1 cells	↓Proliferation	↓ Phosphorylation of AKT and ERK	[30]
3	Gastric cancer	SGC-7901 cells	↓ Proliferation	↑Photodynamic activity & ↑caspase 9, caspase-3 levels	[69]
4	Gastric cancer	AGS, NCI-N87	↑ Apoptosis	↑Caspase-3 and nuclear shrinkage	[70]
5	Gastric cancer	MKN45	↓Proliferation	Cell cycle arrest at G2/M phase and cell death	[71]
6	Gastric cancer	SGC-7901	↓Proliferation and ↑apoptosis	↓Expression of Phosphatase of regenerating-3 (PRL-3)	[72]
7	Colorectal Cancer	HCT116 and CRC mouse model	↓ Proliferation	↓VEGF secretion and VEGFR1, VEGFR2 expression by ↓ACSL4	[41]
8	Colorectal Cancer	HCT116 and Xenograft tumor model	↓Proliferation	↓VEGFR2, PI3K, p-AKT	[73]
9	Colorectal Cancer	SW480 and SW620	↓Cellular proliferation, differentiation and apoptosis	↓ Wnt signalling pathway by ↓TCF/LEF transcriptional activity	[74]
10	Colorectal Cancer	SW480 and SW620	↑Apoptosis	↑ROS by ↑p38/p53 and puma signaling	[75]
11	Pancreatic cancer	SW1990	↑ Apoptosis and ↓metastasis	↓EMT (Epithelial mesenchymal transition) and invasion by ↑miR-1271	[76]
12	Pancreatic cancer	PANC-1	↓Proliferation	↓Methylation of p16, RASSF1A, ppENK	[77]
13	Pancreatic cancer	PANC-1 and BxPC-3	↓Proliferation	EGFR ↑phosphorylation of Stat3	[47]
14	Pancreatic cancer	Sw1990 & Panc-1	↑ Apoptosis and ↓angiogenesis	By ↓ NF-κB and ↓VEGF, MMP-2, MMP-9 & eNOS Phosphorlyation	[78]
15	Liver cancer	HepG2 cells	↓Cellular proliferation	↓ SMAD _2/4_ by ↑miR-34a & ↓VEGFR_2_-AKT-ERK_1/2_	[79]
16	Liver cancer	SMMC-7721	↑ Apoptosis	↑Phosphorylation of ERK, p38 & ↓pAKT	[80]
17	Liver cancer	HepG2 cells	↓ Proliferation and metastasis	↑S and G2/M phase arrest and promotes snail and β-catenin degradation, ↓PI3K/AKT/mTOR	[81]

## Role of nanotechnology and synergism with emodin

The pharmacokinetic limited properties of emodin significantly affect its bioavailability and bioactivity, hence limiting its therapeutic uses. Nanotechnology based drug carriers have been researched to help get past these obstacles. Studies employing phytochemicals in synergism demonstrated better therapeutic opportunity against growth of cancer. EMO exhibits broader range of pharmacological activities and in combination with nano range molecules. Their physiological properties can be enhanced providing combinatorial effect and as targeted therapy. EMO is derivative of anthraquinone and exhibit lower toxicity and appears to be cheaper. For instance, EMO loaded stearic acid—g—chitosan oligosaccharide nano-micelles (CSO-SA-EMO) significantly enhanced antioncogenic properties against the gastric cancer cells. The combinatorial CSO-SA-EMO profoundly arrested the MGC803 and BGC823 cells in G2/M phase of cell cycle. The antitumor effect of CSO-SA-EMO compound was proved by HE staining, tumour volume and TUNEL assay against the gastric cancer cells. It also exhibited upright submissive targeting effect in vivo [82].

Pancreatic cancer is hard to treat due to lack of atypical symptoms. Emodin as natural economic product with magnetic nanoparticles achieves targeted therapy in pancreatic cancer. EMO loaded Cy7-NHS ester activated PEG coated Fe_3_O_4_ (Fe_3_O_4_- PEG-Cy7-EMO) nanoparticles were targeted in vitro and in vivo. The Fe_3_O_4_- PEG-Cy7-EMO nanoparticles showed greater inhibition effect in BxPC-3 cells. The analysis validated that therapeutic effect of Fe_3_O_4_- PEG-Cy7-EMO nanoparticles was greater in BxPC-3 cells than hTERT-HPNE cells. This revealed these nanoparticles combinatorial has more phagocytic ability in pancreatic cancer cells than normal pancreatic cells. The Fe_3_O_4_- PEG-Cy7-EMO nanoparticles also proved potential nanoplatform in pancreatic tumor xenografted mice. Histological analysis of Fe_3_O_4_- PEG-Cy7-EMO nanoparticles was performed in vivo and no pathological changes was observed in heart, liver, kidney and lungs indicates salability to in vivo experiments [83]. Thus, nanoparticles in conjugation with EMO demonstrated their potential towards the gastrointestinal cancers as they facilitate treatment efficiently compared to only EMO or nanoparticles. The number studies were conducted in conjugation with EMO and nanoparticles with greater efficiency against gastrointestinal cancers [84, 85].

## Safety aspects of emodin

Emodin is generally safe medication, based on data from research using experimental animals. For instance, recent study shows that subchronic (12 week) emodin treatment at a maximum dose of 80 mg/Kg body weight orally and 40 mg/Kg body weight intraperitoneally does not significantly harm patients. In mice of both sexes there is no harm on liver, colon, intestine or cardiovascular system [86]. The molecule of emodin is regarded as harmless or safe as due to extensive history of emodin rich plants such as rhubarb and aloe being used by human. Despite of their positive effect on cancers, adverse effect of higher doses are also reported [87]. However, emodin dosage higher than 500–1500 mg/Kg of body weight may cause toxicity such as hepatotoxicity and inflammation [87, 88]. More specifically, it was shown emodin caused Sprague- Dawley rat’ liver to damage because too much of it collected in the liver cells and hindered the movement of FADH or NADH from cytoplasm to mitochondria and results in activation of mitochondrial apoptosis [87]. It is important to acknowledge that majority of emodin toxicity studies had been based upon excessive dosage (far higher than necessary therapeutic levels). Therefore, in-depth evaluations of therapeutic levels of emodin are necessary in both human and experimental animals.

## Recent trends and future prospective

Emodin demonstrates potent anticancer effects by employing multiple mechanisms, including the suppression of cell proliferation, the activation of apoptotic pathways, and the inhibition of tumor angiogenesis and metastatic processes. Literature reported the use of combinatorial method for number of diseases like acute pancreatitis [89], obesity [90], bone disorder [91], cancer [92]. The combinatorial therapy may reduce the chances of drug resistance, side effects and enhances potential of drug. Recent studies exhibited higher potential of use of emodin or emodin analogues with other therapeutic compounds to enhance their efficiency in treatment of various types of cancers [9, 54, 93, 94]. Understanding the intricate relationships between the tumor microenvironment, cancer progression, and immune resistance is crucial for devising optimal therapeutic strategies for all gastrointestinal solid tumors. In the future, it is anticipated that the targeted delivery of emodin could effectively impact gastrointestinal cancer in a predictable and reproducible manner. Additionally, the development of advanced pharmaceutical nanoformulations is crucial to enhance its bioavailability. The translation of anticancer properties of emodin to clinical applications, need further preclincical to clinical studies to evaluate its safety. Emodin is promosing natural compound as therapeutic compound for treatment of GI cancer which warrants further investigation to facilitate development of novel strategy for treatment of GI cancers. Based on current evidence, emodin emerges as a biomolecule with substantial potential for future therapeutic applications.

## Conclusion

Over past years, alternative therapeutic approaches have increased in response to staggering global rise in cancer incidence and mortality rate. There are tireless efforts made to extend reach of traditional medical procedures such as radiation, chemotherapy, immunotherapy and surgical but global study of nutraceuticals made the impact. Recent research studies have shown significant impression of emodin on progress of GI cancers. The number of studies demonstrated the emodin inhibits the pathways that lead to inflammation, angiogenesis, proliferation, tumorigenesis in addition to mitochondrial mediated apoptosis. Recent research targeting tumor progression in vivo and in vitro has shown encouraging results using emodin. The profile of emodin showed metabolism and absorption of lower doses with not significant toxicity. The intriguing aspect of potential of emodin as a supplementary treatment to traditional chemotherapeutics is its capacity to enhance the efficiency of nanoparticles. In conclusion emodin should be further explored in this area as it has lot of potential to treat GI cancers. Emodin can be used alone or it can be used in combination with nanoparticles to treat GI cancers. In broader sense collaborative research in fields of pharmacology, toxicology, pharmacokinetics and pharmaceutics is required to attain safety and specificity for successful GI treatment.

## Supplementary Information

Supplementary material 1.

## Data Availability

No datasets were generated or analysed during the current study.

## Abbreviations

CRC: Colorectal cancer
EMT: Epithelial-mesenchymal transition
EC: Esophageal cancer
GC: Gastric cancer
GI: Gastrointestinal cancer
miRNA 1271: Micro-RNA-1271
MMA: Mitochondrial mediate apoptosis
OKS: Octaketide synthase
PC: Pancreatic cancer
ROS: Reactive oxygen species
PKS: Type III polyketide synthase
VEGFR2: Vascular endothelial growth factor receptor 2
