# Supplementary figures and images for: Role of emodin to prevent gastrointestinal cancers: recent trends and future prospective
Source: Discov Oncol. 2025 Apr 5;16:468. doi: 10.1007/s12672-025-02240-9 (PMC11972247; doi:10.1007/s12672-025-02240-9)

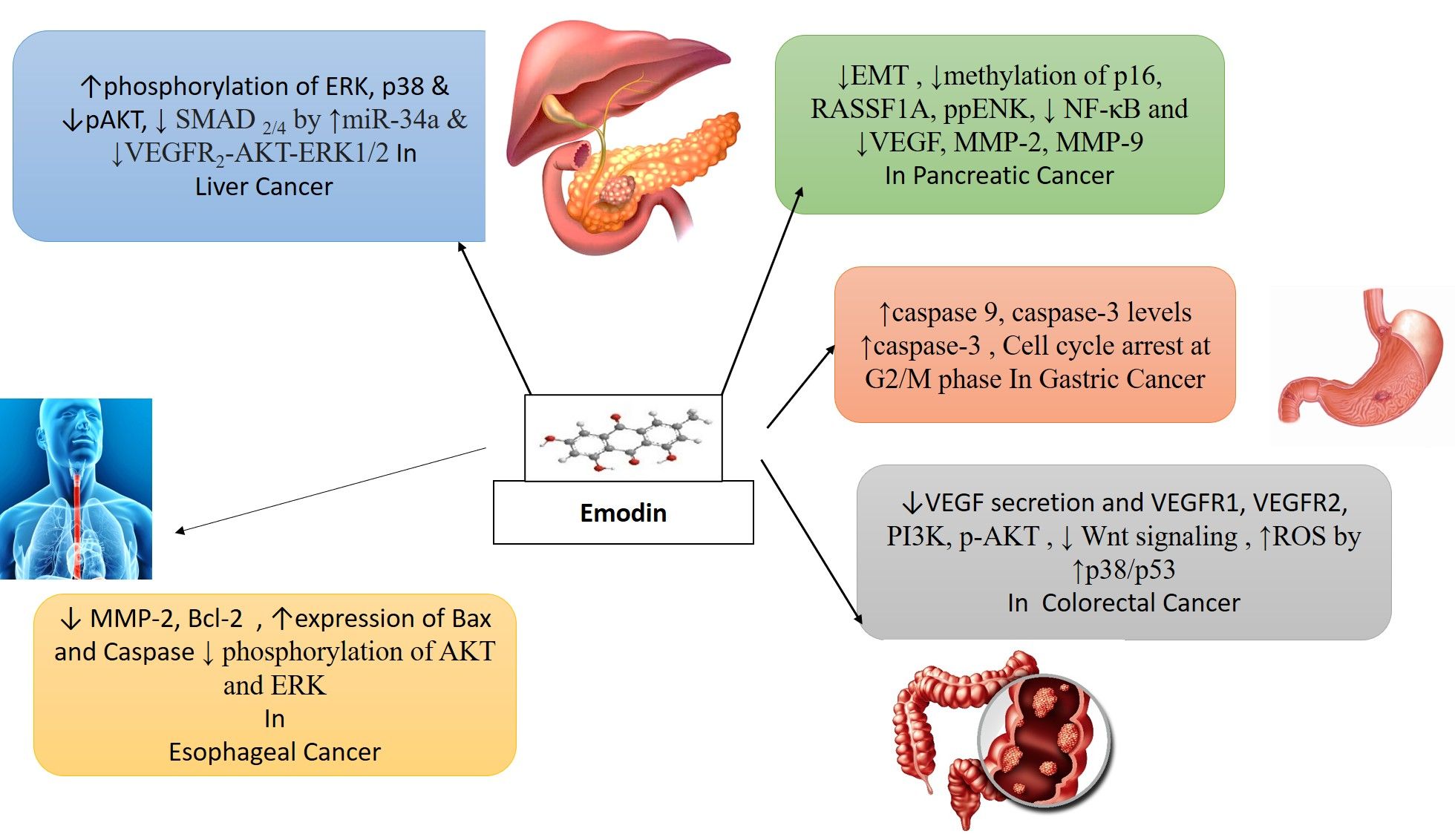

Supplement: Supplementary file 1 — Supplementary material 1. [file 12672_2025_2240_MOESM1_ESM.jpg]
